# Supplementary material for: COZOID: contact zone identifier for visual analysis of protein-protein interactions
Source: BMC Bioinformatics. 2018 Apr 6;19:125. doi: 10.1186/s12859-018-2113-6 (PMC5889581; doi:10.1186/s12859-018-2113-6)
Supplement: Supplementary file 2 — Software build. Executable binary file of the software tool. (ZIP 118,784 kb) [file 12859_2018_2113_MOESM2_ESM.zip › cozoid/java/docs/beansbinding-1.2.1-doc.zip!/org/jdesktop/el/impl/parser/class-use/Token.html]

Uses of Class org.jdesktop.el.impl.parser.Token (Beans Binding)


---


|  |  |  |
| --- | --- | --- |
| **Overview** \| **Package** \| **Class** \| **Use** \| **Tree** \| **Deprecated** \| **Index** \| **Help** | |  |
| PREV   NEXT | **FRAMES**    **NO FRAMES**     **All Classes** |  |


---


## **Uses of Class org.jdesktop.el.impl.parser.Token**

| Packages that use Token | |
| --- | --- |
| **org.jdesktop.el.impl.parser** | Customized version of EL for Beans Binding; not for general use. |

Uses of Token in org.jdesktop.el.impl.parser

| Fields in org.jdesktop.el.impl.parser declared as Token | |
| --- | --- |
| `Token` | `ParseException.currentToken`             This is the last token that has been consumed successfully. |
| `Token` | `ELParser.jj_nt` |
| `Token` | `Token.next`             A reference to the next regular (non-special) token from the input stream. |
| `Token` | `Token.specialToken`             This field is used to access special tokens that occur prior to this token, but after the immediately preceding regular (non-special) token. |
| `Token` | `ELParser.token` |

| Methods in org.jdesktop.el.impl.parser that return Token | |
| --- | --- |
| `Token` | `ELParserTokenManager.getNextToken()` |
| `Token` | `ELParser.getNextToken()` |
| `Token` | `ELParser.getToken(int index)` |
| `protected  Token` | `ELParserTokenManager.jjFillToken()` |
| `static Token` | `Token.newToken(int ofKind)`             Returns a new Token object, by default. |

Constructors in org.jdesktop.el.impl.parser with parameters of type Token

`ParseException(Token currentTokenVal,
int[][] expectedTokenSequencesVal,
java.lang.String[] tokenImageVal)`
  
          This constructor is used by the method "generateParseException"
in the generated parser.

---


|  |  |  |
| --- | --- | --- |
| **Overview** \| **Package** \| **Class** \| **Use** \| **Tree** \| **Deprecated** \| **Index** \| **Help** | |  |
| PREV   NEXT | **FRAMES**    **NO FRAMES**     **All Classes** |  |


---
